# Detecting differential expression in microarray data: comparison of optimal procedures

**DOI:** 10.1186/1471-2105-8-28

**Published:** 2007-01-26

**Authors:** Elena Perelman, Alexander Ploner, Stefano Calza, Yudi Pawitan

**Affiliations:** 1 Department of Medical Epidemiology and Biostatistics, Karolinska Institutet, 17177 Stockholm, Sweden; 2 Department of Biomedical Sciences and Biotechnologies, Brescia, Italy

## Abstract

**Background:**

Many procedures for finding differentially expressed genes in microarray data are based on classical or modified t-statistics. Due to multiple testing considerations, the false discovery rate (FDR) is the key tool for assessing the significance of these test statistics. Two recent papers have generalized two aspects: Storey et al. (2005) have introduced a likelihood ratio test statistic for two-sample situations that has desirable theoretical properties (optimal discovery procedure, ODP), but uses standard FDR assessment; Ploner et al. (2006) have introduced a multivariate local FDR that allows incorporation of standard error information, but uses the standard t-statistic (fdr2d). The relationship and relative performance of these methods in two-sample comparisons is currently unknown.

**Methods:**

Using simulated and real datasets, we compare the ODP and fdr2d procedures. We also introduce a new procedure called S2d that combines the ODP test statistic with the extended FDR assessment of fdr2d.

**Results:**

For both simulated and real datasets, fdr2d performs better than ODP. As expected, both methods perform better than a standard t-statistic with standard local FDR. The new procedure S2d performs as well as fdr2d on simulated data, but performs better on the real data sets.

**Conclusion:**

The ODP can be improved by including the standard error information as in fdr2d. This means that the optimality enjoyed in theory by ODP does not hold for the estimated version that has to be used in practice. The new procedure S2d has a slight advantage over fdr2d, which has to be balanced against a significantly higher computational effort and a less intuititive test statistic.

## Background

High-throughput methods in molecular biology have challenged existing data analysis methods and stimulated the development of new methods. A key example is the gene expression microarray and its use as a screening tool for detecting genes that are differentially expressed (DE) between different biological states. The need to identify a possibly very small number of regulated genes among the 10,000s of sequences found on modern microarray chips, based on tens to hundreds of biological samples, has led to a plethora of different methods. The emerging consensus in the field [1] suggests that a) despite ongoing research on p-value adjustments [2], false discovery rates (FDR, [3]) are more practical for dealing with the multiplicity problem, and b) classical test statistics requires modification to limit the influence of unrealistically small variance estimates. Nonetheless, many competing methods for detecting DE exist, and even attempts at validation on data sets with known mRNA composition [4] cannot offer definitive guidelines.

In this context, the introduction of the so-called optimal discovery procedure (ODP, [5]) constitutes a major conceptual achievement. Building on the Neyman-Pearson lemma for testing an individual hypothesis, the author shows that an extension of the likelihood ratio test statistic for multiple parallel hypotheses (or genes) is the optimal procedure for deciding whether any specific gene is in fact DE: for any fixed number of false positive results, ODP will identify the maximum number of true positives. The ODP establishes therefore a theoretical optimum for detecting DE against which any other method can be measured.

Unfortunately, the optimality of ODP is a strictly theoretical result that requires, for all genes, a full parametric specification of the densities under null and alternative hypothesis. In practice, even assuming normality, the gene-wise means and variances are unknown, and they become nuisance parameters in the hypothesis testing. Consequently, the authors of [6] have suggested an estimated version EODP, which can be implemented in practice. It is, however, not clear how EODP performs compared to the theoretical optimum, or other existing methods, except under the most benign circumstances (no correlation and equal variances between genes).

The main questions of this paper are therefore a) whether the optimality of ODP is retained by EODP, and b) whether we can improve on EODP's performance in practice. Previously, we have introduced a multidimensional extension of the FDR procedure (fdr2d) that combines standard error information with the classical t-statistic. We demonstrated that the fdr2d performs as well or better than the usual modified t-statistics, without requiring extra modeling or model assumptions [7]. In this paper, we show that fdr2d also outperforms EODP on simulated and real data sets. We also demonstrate how a synthesis of the EODP and fdr2d procedures can further improve the power to detect DE.

### The two-sample problem

We demonstrate the application of EODP and fdr2d in the common situation where we want to detect genes that are DE between two biological states. We assume *n*_1 _and *n*_2 _arrays for each group, each containing probes for *m *genes. For gene *i*, we observe a vector of expression values **x_i _**of length *n*_1 _+ *n*_2_, which consists of the observations **x_i1 _**in the first group, and **x_i2 _**in the second group. We define the groupwise means and standard deviations as usual, and refer to the pooled standard deviation as

s˜i2=(n1−1)si12+(n2−1)si22n1+n2−2.
 MathType@MTEF@5@5@+=feaafiart1ev1aaatCvAUfKttLearuWrP9MDH5MBPbIqV92AaeXatLxBI9gBaebbnrfifHhDYfgasaacH8akY=wiFfYdH8Gipec8Eeeu0xXdbba9frFj0=OqFfea0dXdd9vqai=hGuQ8kuc9pgc9s8qqaq=dirpe0xb9q8qiLsFr0=vr0=vr0dc8meaabaqaciaacaGaaeqabaqabeGadaaakeaacuWGZbWCgaacamaaDaaaleaacqWGPbqAaeaacqaIYaGmaaGccqGH9aqpdaWcaaqaaiabcIcaOiabd6gaUnaaBaaaleaacqaIXaqmaeqaaOGaeyOeI0IaeGymaeJaeiykaKIaem4Cam3aa0baaSqaaiabdMgaPjabigdaXaqaaiabikdaYaaakiabgUcaRiabcIcaOiabd6gaUnaaBaaaleaacqaIYaGmaeqaaOGaeyOeI0IaeGymaeJaeiykaKIaem4Cam3aa0baaSqaaiabdMgaPjabikdaYaqaaiabikdaYaaaaOqaaiabd6gaUnaaBaaaleaacqaIXaqmaeqaaOGaey4kaSIaemOBa42aaSbaaSqaaiabikdaYaqabaGccqGHsislcqaIYaGmaaGaeiOla4caaa@5161@

Furthermore, we assume that we are dealing with a random mixture of DE and nonDE genes, with a proportion *π*_0 _of genes being nonDE.

### ODP statistics

The theoretical ODP statistic assumes that for all *i *= 1, ... *m *genes, the density functions of the expression values under the null hypothesis of no DE, *f*_*i*_, and under the alternative hypothesis of DE, *g*_*i*_, are fully known in advance. For the random mixture of DE and nonDE genes outlined above, the ODP statistic for the observed expression values **x_i _**of the *i*-the gene can then be written as

SODP(xi)=∑j=1mgj(xi)∑j=1mfj(xi).
 MathType@MTEF@5@5@+=feaafiart1ev1aaatCvAUfKttLearuWrP9MDH5MBPbIqV92AaeXatLxBI9gBaebbnrfifHhDYfgasaacH8akY=wiFfYdH8Gipec8Eeeu0xXdbba9frFj0=OqFfea0dXdd9vqai=hGuQ8kuc9pgc9s8qqaq=dirpe0xb9q8qiLsFr0=vr0=vr0dc8meaabaqaciaacaGaaeqabaqabeGadaaakeaacqWGtbWudaWgaaWcbaGaee4ta8KaeeiraqKaeeiuaafabeaakiabcIcaOGqabiab=Hha4naaBaaaleaacqWFPbqAaeqaaOGaeiykaKIaeyypa0ZaaSaaaeaadaaeWaqaaiabdEgaNnaaBaaaleaacqWGQbGAaeqaaOGaeiikaGIae8hEaG3aaSbaaSqaaiab=LgaPbqabaGccqGGPaqkaSqaaiabdQgaQjabg2da9iabigdaXaqaaiabd2gaTbqdcqGHris5aaGcbaWaaabmaeaacqWGMbGzdaWgaaWcbaGaemOAaOgabeaakiabcIcaOiab=Hha4naaBaaaleaacqWFPbqAaeqaaOGaeiykaKcaleaacqWGQbGAcqGH9aqpcqaIXaqmaeaacqWGTbqBa0GaeyyeIuoaaaGccqGGUaGlaaa@54E2@

The procedure then rejects the null hypothesis for all genes *i *with *S*_*i *_≡ *S*(**x_i_**) ≥ *λ*, i.e. all genes with large *S*_*i *_are declared to be DE. Using the Neyman-Pearson Lemma, it can be shown that this procedure is optimal in the sense that for any pre-specified false positive rate (which will determine *λ*), the ODP will have the maximum true positive rate. This optimality property can also be expressed in terms of FDR [5].

Requiring full specification of all null and alternative distributions, however, is impractical. In any realistic application, only an estimated ODP statistic

S^ODP(xi)=∑j=1mg^j(xi)∑j=1mf^j(xi)
 MathType@MTEF@5@5@+=feaafiart1ev1aaatCvAUfKttLearuWrP9MDH5MBPbIqV92AaeXatLxBI9gBaebbnrfifHhDYfgasaacH8akY=wiFfYdH8Gipec8Eeeu0xXdbba9frFj0=OqFfea0dXdd9vqai=hGuQ8kuc9pgc9s8qqaq=dirpe0xb9q8qiLsFr0=vr0=vr0dc8meaabaqaciaacaGaaeqabaqabeGadaaakeaacuWGtbWugaqcamaaBaaaleaacqqGpbWtcqqGebarcqqGqbauaeqaaOGaeiikaGccbeGae8hEaG3aaSbaaSqaaiab=LgaPbqabaGccqGGPaqkcqGH9aqpdaWcaaqaamaaqadabaGafm4zaCMbaKaadaWgaaWcbaGaemOAaOgabeaakiabcIcaOiab=Hha4naaBaaaleaacqWFPbqAaeqaaOGaeiykaKcaleaacqWGQbGAcqGH9aqpcqaIXaqmaeaacqWGTbqBa0GaeyyeIuoaaOqaamaaqadabaGafmOzayMbaKaadaWgaaWcbaGaemOAaOgabeaakiabcIcaOiab=Hha4naaBaaaleaacqWFPbqAaeqaaOGaeiykaKcaleaacqWGQbGAcqGH9aqpcqaIXaqmaeaacqWGTbqBa0GaeyyeIuoaaaaaaa@5424@

is feasible, where the densities f^i
 MathType@MTEF@5@5@+=feaafiart1ev1aaatCvAUfKttLearuWrP9MDH5MBPbIqV92AaeXatLxBI9gBaebbnrfifHhDYfgasaacH8akY=wiFfYdH8Gipec8Eeeu0xXdbba9frFj0=OqFfea0dXdd9vqai=hGuQ8kuc9pgc9s8qqaq=dirpe0xb9q8qiLsFr0=vr0=vr0dc8meaabaqaciaacaGaaeqabaqabeGadaaakeaacuWGMbGzgaqcamaaBaaaleaacqWGPbqAaeqaaaaa@2F98@ and g^i
 MathType@MTEF@5@5@+=feaafiart1ev1aaatCvAUfKttLearuWrP9MDH5MBPbIqV92AaeXatLxBI9gBaebbnrfifHhDYfgasaacH8akY=wiFfYdH8Gipec8Eeeu0xXdbba9frFj0=OqFfea0dXdd9vqai=hGuQ8kuc9pgc9s8qqaq=dirpe0xb9q8qiLsFr0=vr0=vr0dc8meaabaqaciaacaGaaeqabaqabeGadaaakeaacuWGNbWzgaqcamaaBaaaleaacqWGPbqAaeqaaaaa@2F9A@ are estimated from the data. In [6], the authors propose to assume that all genes follow a normal distribution (possibly after suitable transformation); under this assumption, only means and variances have to be estimated from the data. In our two-sample situation, this amounts to

S^ODP(xi)∑j=1mφ(xi1|μ^j1,σ^j12)φ(xi2|μ^j2,σ^j12)∑j=1mφ(xi|μ^j,σ^j02)     (1)
 MathType@MTEF@5@5@+=feaafiart1ev1aaatCvAUfKttLearuWrP9MDH5MBPbIqV92AaeXatLxBI9gBaebbnrfifHhDYfgasaacH8akY=wiFfYdH8Gipec8Eeeu0xXdbba9frFj0=OqFfea0dXdd9vqai=hGuQ8kuc9pgc9s8qqaq=dirpe0xb9q8qiLsFr0=vr0=vr0dc8meaabaqaciaacaGaaeqabaqabeGadaaakeaacuWGtbWugaqcamaaBaaaleaacqqGpbWtcqqGebarcqqGqbauaeqaaOGaeiikaGccbeGae8hEaG3aaSbaaSqaaiab=LgaPbqabaGccqGGPaqkdaWcaaqaamaaqadabaacciGae4NXdy2aaeWaaeaacqWF4baEdaWgaaWcbaGae8xAaKMae8xmaedabeaakiabcYha8jqb+X7aTzaajaWaaSbaaSqaaiabdQgaQjabigdaXaqabaGccqGGSaalcuGFdpWCgaqcamaaDaaaleaacqWGQbGAcqaIXaqmaeaacqaIYaGmaaaakiaawIcacaGLPaaacqGFgpGzdaqadaqaaiab=Hha4naaBaaaleaacqWFPbqAcqWFYaGmaeqaaOGaeiiFaWNaf4hVd0MbaKaadaWgaaWcbaGaemOAaOMaeGOmaidabeaakiabcYcaSiqb+n8aZzaajaWaa0baaSqaaiabdQgaQjabigdaXaqaaiabikdaYaaaaOGaayjkaiaawMcaaaWcbaGaemOAaOMaeyypa0JaeGymaedabaGaemyBa0ganiabggHiLdaakeaadaaeWaqaaiab+z8aMnaabmaabaGae8hEaG3aaSbaaSqaaiab=LgaPbqabaGccqGG8baFcuGF8oqBgaqcamaaBaaaleaacqWGQbGAaeqaaOGaeiilaWIaf43WdmNbaKaadaqhaaWcbaGaemOAaOMaeGimaadabaGaeGOmaidaaaGccaGLOaGaayzkaaaaleaacqWGQbGAcqGH9aqpcqaIXaqmaeaacqWGTbqBa0GaeyyeIuoaaaGccaWLjaGaaCzcamaabmaabaGaeGymaedacaGLOaGaayzkaaaaaa@7EE3@

where *φ*(·|*μ*, *σ*^2^) is the joint-density for the normal distribution with mean *μ *and variance *σ*^2^.

Conceptually, under the null hypothesis, we have the usual estimates μ^j=x¯j
 MathType@MTEF@5@5@+=feaafiart1ev1aaatCvAUfKttLearuWrP9MDH5MBPbIqV92AaeXatLxBI9gBaebbnrfifHhDYfgasaacH8akY=wiFfYdH8Gipec8Eeeu0xXdbba9frFj0=OqFfea0dXdd9vqai=hGuQ8kuc9pgc9s8qqaq=dirpe0xb9q8qiLsFr0=vr0=vr0dc8meaabaqaciaacaGaaeqabaqabeGadaaakeaaiiGacuWF8oqBgaqcamaaBaaaleaacqWGQbGAaeqaaOGaeyypa0JafmiEaGNbaebadaWgaaWcbaGaemOAaOgabeaaaaa@342C@ and σ^j02=sj2
 MathType@MTEF@5@5@+=feaafiart1ev1aaatCvAUfKttLearuWrP9MDH5MBPbIqV92AaeXatLxBI9gBaebbnrfifHhDYfgasaacH8akY=wiFfYdH8Gipec8Eeeu0xXdbba9frFj0=OqFfea0dXdd9vqai=hGuQ8kuc9pgc9s8qqaq=dirpe0xb9q8qiLsFr0=vr0=vr0dc8meaabaqaciaacaGaaeqabaqabeGadaaakeaaiiGacuWFdpWCgaqcamaaDaaaleaacqWGQbGAcqaIWaamaeaacqaIYaGmaaGccqGH9aqpcqWGZbWCdaqhaaWcbaGaemOAaOgabaGaeGOmaidaaaaa@36EB@ from the combined data, and under the alternative hypothesis, the corresponding group-wise means μ^j1=x¯j1
 MathType@MTEF@5@5@+=feaafiart1ev1aaatCvAUfKttLearuWrP9MDH5MBPbIqV92AaeXatLxBI9gBaebbnrfifHhDYfgasaacH8akY=wiFfYdH8Gipec8Eeeu0xXdbba9frFj0=OqFfea0dXdd9vqai=hGuQ8kuc9pgc9s8qqaq=dirpe0xb9q8qiLsFr0=vr0=vr0dc8meaabaqaciaacaGaaeqabaqabeGadaaakeaaiiGacuWF8oqBgaqcamaaBaaaleaacqWGQbGAcqaIXaqmaeqaaOGaeyypa0JafmiEaGNbaebadaWgaaWcbaGaemOAaOMaeGymaedabeaaaaa@360C@ and μ^j2=x¯j2
 MathType@MTEF@5@5@+=feaafiart1ev1aaatCvAUfKttLearuWrP9MDH5MBPbIqV92AaeXatLxBI9gBaebbnrfifHhDYfgasaacH8akY=wiFfYdH8Gipec8Eeeu0xXdbba9frFj0=OqFfea0dXdd9vqai=hGuQ8kuc9pgc9s8qqaq=dirpe0xb9q8qiLsFr0=vr0=vr0dc8meaabaqaciaacaGaaeqabaqabeGadaaakeaaiiGacuWF8oqBgaqcamaaBaaaleaacqWGQbGAcqaIYaGmaeqaaOGaeyypa0JafmiEaGNbaebadaWgaaWcbaGaemOAaOMaeGOmaidabeaaaaa@3610@ with the pooled sample variance σ^j12=s˜j2
 MathType@MTEF@5@5@+=feaafiart1ev1aaatCvAUfKttLearuWrP9MDH5MBPbIqV92AaeXatLxBI9gBaebbnrfifHhDYfgasaacH8akY=wiFfYdH8Gipec8Eeeu0xXdbba9frFj0=OqFfea0dXdd9vqai=hGuQ8kuc9pgc9s8qqaq=dirpe0xb9q8qiLsFr0=vr0=vr0dc8meaabaqaciaacaGaaeqabaqabeGadaaakeaaiiGacuWFdpWCgaqcamaaDaaaleaacqWGQbGAcqaIXaqmaeaacqaIYaGmaaGccqGH9aqpcuWGZbWCgaacamaaDaaaleaacqWGQbGAaeaacqaIYaGmaaaaaa@36FC@. For the practical implementation, we follow [6] and pre-normalize all genes to have zero mean.

The second step in applying the ODP to data is the calibration of the procedure. There is no distribution theory for the statistic, so it is not clear how to choose the threshold λ to achieve a desired FDR level. [6] suggest a conventional algorithm that computes the estimated ODP statistic S^
 MathType@MTEF@5@5@+=feaafiart1ev1aaatCvAUfKttLearuWrP9MDH5MBPbIqV92AaeXatLxBI9gBaebbnrfifHhDYfgasaacH8akY=wiFfYdH8Gipec8Eeeu0xXdbba9frFj0=OqFfea0dXdd9vqai=hGuQ8kuc9pgc9s8qqaq=dirpe0xb9q8qiLsFr0=vr0=vr0dc8meaabaqaciaacaGaaeqabaqabeGadaaakeaacuWGtbWugaqcaaaa@2DEB@ under random permutations of the group labels; they use the resulting null distribution of S^
 MathType@MTEF@5@5@+=feaafiart1ev1aaatCvAUfKttLearuWrP9MDH5MBPbIqV92AaeXatLxBI9gBaebbnrfifHhDYfgasaacH8akY=wiFfYdH8Gipec8Eeeu0xXdbba9frFj0=OqFfea0dXdd9vqai=hGuQ8kuc9pgc9s8qqaq=dirpe0xb9q8qiLsFr0=vr0=vr0dc8meaabaqaciaacaGaaeqabaqabeGadaaakeaacuWGtbWugaqcaaaa@2DEB@ to compute the q-value for each gene, which represents its global FDR (e.g. [8]). We follow this approach for our implementation, but use the local false discovery rate (fdr, see [9] and below), with essentially identical results as theirs.

### Multidimensional local false discovery rate

FDR approaches focus on the distribution of the specific statistic *Z *used to test the gene-wise null hypotheses, in contrast to ODP, which is based on the distribution of the data. Given a mixture of DE and nonDE genes as described above, the density *f *of *Z *can be written as

*f*(*z*) = *π*_0_*f*_0_(*z*) + (1 - *π*_0_)*f*_1_(*z*),     (2)

where *f*_0 _and *f*_1 _are the densities of the test statistic *Z *for nonDE and DE genes, respectively, and *π*_0 _the proportion of truly nonDE genes. The local fdr for any observed value *z *of the test statistic is then

fdr(z)=π0f0(z)f(z),     (3)
 MathType@MTEF@5@5@+=feaafiart1ev1aaatCvAUfKttLearuWrP9MDH5MBPbIqV92AaeXatLxBI9gBaebbnrfifHhDYfgasaacH8akY=wiFfYdH8Gipec8Eeeu0xXdbba9frFj0=OqFfea0dXdd9vqai=hGuQ8kuc9pgc9s8qqaq=dirpe0xb9q8qiLsFr0=vr0=vr0dc8meaabaqaciaacaGaaeqabaqabeGadaaakeaacqqGMbGzcqqGKbazcqqGYbGCcqGGOaakcqWG6bGEcqGGPaqkcqGH9aqpiiGacqWFapaCdaWgaaWcbaGaeGimaadabeaakmaalaaabaGaemOzay2aaSbaaSqaaiabicdaWaqabaGccqGGOaakcqWG6bGEcqGGPaqkaeaacqWGMbGzcqGGOaakcqWG6bGEcqGGPaqkaaGaeiilaWIaaCzcaiaaxMaadaqadaqaaiabiodaZaGaayjkaiaawMcaaaaa@46B3@

and can be interpreted as the expected rate of false positives among genes with test statistic *z*, see [9]. Practically, the densities *f *can be estimated from the histograms of the test statistics computed from the real data, and *f*_0 _is estimated similarly from the test statistics computed from permuted data.

Formulated as a decision procedure like ODP, we specify a test statistic *Z *and a desired threshold *α *for the local fdr; we then compute for each gene the value of the test statistic *z*_*i *_= *Z*(**x_i_**) and the decision criterion fdr_*i *_= fdr(*z*_*i*_) and declare genes with fdr_*i *_<*α *to be DE.

As the more usual global FDR of a set of test statistics is just the average of their local fdr [9], little seems to be gained by using the local fdr. Note, however, that Equations (2) and (3) still hold if we replace the univariate test statistic *Z *by a vector **Z **of test statistics. We have recently shown that for the two-sample problem, using a bivariate test statistic and the associated two-dimensional fdr is more powerful than conventional FDR for univariate test statistics [7]. Specifically, the test statistic **Z **= (*Z*_1_, *Z*_2_) with

*Z*_1 _= *t *and *Z*_2 _= log *se*,     (4)

where *t *is the usual t statistic, and *se *the standard error of the mean,

se=s˜1/n1+1/n2,
 MathType@MTEF@5@5@+=feaafiart1ev1aaatCvAUfKttLearuWrP9MDH5MBPbIqV92AaeXatLxBI9gBaebbnrfifHhDYfgasaacH8akY=wiFfYdH8Gipec8Eeeu0xXdbba9frFj0=OqFfea0dXdd9vqai=hGuQ8kuc9pgc9s8qqaq=dirpe0xb9q8qiLsFr0=vr0=vr0dc8meaabaqaciaacaGaaeqabaqabeGadaaakeaacqWGZbWCcqWGLbqzcqGH9aqpcuWGZbWCgaacamaakaaabaGaeGymaeJaei4la8IaemOBa42aaSbaaSqaaiabigdaXaqabaGccqGHRaWkcqaIXaqmcqGGVaWlcqWGUbGBdaWgaaWcbaGaeGOmaidabeaaaeqaaOGaeiilaWcaaa@3C88@

yields smaller fdr not only compared to the conventional t-statistic on its own, but also compared to a number of popular modified t-statistics [10-12].

In the following, we will use the abbreviations fdr1d and fdr2d for local fdr computed based on univariate and bivariate test statistics, respectively. Note that in practice, the fdr2d is estimated in a similar manner as the fdr1d, using two-dimensional histograms instead of one-dimensional histograms, together with a somewhat more sophisticated binomial smoothing procedure, see [7] for details.

### Procedures to be evaluated

The central aim of this paper is to compare the operating characteristics of four different procedures for detecting DE on a number of real and simulated data sets:

1. t1d uses the standard t-statistic with conventional fdr1d and serves as a reference.

2. S1d uses the logarithm of S^
 MathType@MTEF@5@5@+=feaafiart1ev1aaatCvAUfKttLearuWrP9MDH5MBPbIqV92AaeXatLxBI9gBaebbnrfifHhDYfgasaacH8akY=wiFfYdH8Gipec8Eeeu0xXdbba9frFj0=OqFfea0dXdd9vqai=hGuQ8kuc9pgc9s8qqaq=dirpe0xb9q8qiLsFr0=vr0=vr0dc8meaabaqaciaacaGaaeqabaqabeGadaaakeaacuWGtbWugaqcaaaa@2DEB@ in (1) with fdr1d; this procedure is equivalent to the estimated version of ODP described in [6] and its implementation in the EDGE software.

3. t2d uses the test statistic in (4) for calculating fdr2d; this is the same procedure as described in [7].

4. S2d is a novel procedure that combines the logarithm of S^
 MathType@MTEF@5@5@+=feaafiart1ev1aaatCvAUfKttLearuWrP9MDH5MBPbIqV92AaeXatLxBI9gBaebbnrfifHhDYfgasaacH8akY=wiFfYdH8Gipec8Eeeu0xXdbba9frFj0=OqFfea0dXdd9vqai=hGuQ8kuc9pgc9s8qqaq=dirpe0xb9q8qiLsFr0=vr0=vr0dc8meaabaqaciaacaGaaeqabaqabeGadaaakeaacuWGtbWugaqcaaaa@2DEB@ and the standard error for computing fdr2d, see below.

## Results

### Feasibility of S2d

We first evaluate the S2d procedure, based on the bivariate test statistic

*Z*_1 _= logS^
 MathType@MTEF@5@5@+=feaafiart1ev1aaatCvAUfKttLearuWrP9MDH5MBPbIqV92AaeXatLxBI9gBaebbnrfifHhDYfgasaacH8akY=wiFfYdH8Gipec8Eeeu0xXdbba9frFj0=OqFfea0dXdd9vqai=hGuQ8kuc9pgc9s8qqaq=dirpe0xb9q8qiLsFr0=vr0=vr0dc8meaabaqaciaacaGaaeqabaqabeGadaaakeaacuWGtbWugaqcaaaa@2DEB@     and     *Z*_2 _= log *se*,

with S^
 MathType@MTEF@5@5@+=feaafiart1ev1aaatCvAUfKttLearuWrP9MDH5MBPbIqV92AaeXatLxBI9gBaebbnrfifHhDYfgasaacH8akY=wiFfYdH8Gipec8Eeeu0xXdbba9frFj0=OqFfea0dXdd9vqai=hGuQ8kuc9pgc9s8qqaq=dirpe0xb9q8qiLsFr0=vr0=vr0dc8meaabaqaciaacaGaaeqabaqabeGadaaakeaacuWGtbWugaqcaaaa@2DEB@ defined as in (1) and *se *as in (4). The only practical concern is that the smoothing procedure described in [7] may have problems with S^
 MathType@MTEF@5@5@+=feaafiart1ev1aaatCvAUfKttLearuWrP9MDH5MBPbIqV92AaeXatLxBI9gBaebbnrfifHhDYfgasaacH8akY=wiFfYdH8Gipec8Eeeu0xXdbba9frFj0=OqFfea0dXdd9vqai=hGuQ8kuc9pgc9s8qqaq=dirpe0xb9q8qiLsFr0=vr0=vr0dc8meaabaqaciaacaGaaeqabaqabeGadaaakeaacuWGtbWugaqcaaaa@2DEB@. Indeed, the reason for taking the logarithms of the test statistics is to facilitate smoothing, by avoiding crowding at the boundary values.

**Figure 1:**
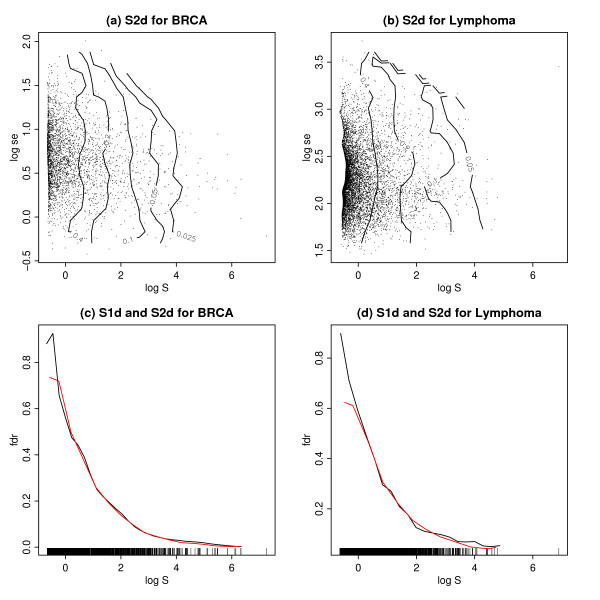
S2d and S1d for the BRCA and Lymphoma data sets. (a) A scatter plot of the BRCA data, with log S^
 MathType@MTEF@5@5@+=feaafiart1ev1aaatCvAUfKttLearuWrP9MDH5MBPbIqV92AaeXatLxBI9gBaebbnrfifHhDYfgasaacH8akY=wiFfYdH8Gipec8Eeeu0xXdbba9frFj0=OqFfea0dXdd9vqai=hGuQ8kuc9pgc9s8qqaq=dirpe0xb9q8qiLsFr0=vr0=vr0dc8meaabaqaciaacaGaaeqabaqabeGadaaakeaacuWGtbWugaqcaaaa@2DEB@ on the horizontal axis and log *se *on the vertical axis. Each symbol corresponds to a gene. The isolines shown are the local fdr based on the S2d method. (b) The same as (a) for the Lymphoma data. (c) The local fdr for the BRCA data, shown as a function of log S^
 MathType@MTEF@5@5@+=feaafiart1ev1aaatCvAUfKttLearuWrP9MDH5MBPbIqV92AaeXatLxBI9gBaebbnrfifHhDYfgasaacH8akY=wiFfYdH8Gipec8Eeeu0xXdbba9frFj0=OqFfea0dXdd9vqai=hGuQ8kuc9pgc9s8qqaq=dirpe0xb9q8qiLsFr0=vr0=vr0dc8meaabaqaciaacaGaaeqabaqabeGadaaakeaacuWGtbWugaqcaaaa@2DEB@ on the horizontal axis. The black line shows the local fdr computed via S1d, the red line shows the fdr based on S2d, averaged across the log standard errors as shown in (a) above. (d) The same as (c) for the Lymphoma data.

Figures 1(a) and 1(b) show the scatter plot of the bivariate test statistics for two real data sets described in Methods, with the estimated fdr2d overlayed as isolines. We exploit the useful fact that we can always average the fdr2d over one of the component statistics to get the fdr1d for the other component statistic:

fdr1d(log⁡S^)=∫fdr2d(log⁡S^,log⁡se)dlog⁡se,
 MathType@MTEF@5@5@+=feaafiart1ev1aaatCvAUfKttLearuWrP9MDH5MBPbIqV92AaeXatLxBI9gBaebbnrfifHhDYfgasaacH8akY=wiFfYdH8Gipec8Eeeu0xXdbba9frFj0=OqFfea0dXdd9vqai=hGuQ8kuc9pgc9s8qqaq=dirpe0xb9q8qiLsFr0=vr0=vr0dc8meaabaqaciaacaGaaeqabaqabeGadaaakeaacqqGMbGzcqqGKbazcqqGYbGCcqaIXaqmcqqGKbazcqGGOaakcyGGSbaBcqGGVbWBcqGGNbWzcuWGtbWugaqcaiabcMcaPiabg2da9maapeaabaGaeeOzayMaeeizaqMaeeOCaiNaeGOmaiJaeeizaqMaeiikaGIagiiBaWMaei4Ba8Maei4zaCMafm4uamLbaKaacqGGSaalcyGGSbaBcqGGVbWBcqGGNbWzcqWGZbWCcqWGLbqzcqGGPaqkcqWGKbazcyGGSbaBcqGGVbWBcqGGNbWzcqWGZbWCcqWGLbqzcqGGSaalaSqabeqaniabgUIiYdaaaa@5B36@

see [7]. Figures 1(c) and 1(d) show S1d (black) overlayed with the averaged S2d (red) for both data sets, with excellent agreement. This indicates that the smoothing required for computing S2d has been successful. This is consistent with the relationship between t-statistics and logS^
 MathType@MTEF@5@5@+=feaafiart1ev1aaatCvAUfKttLearuWrP9MDH5MBPbIqV92AaeXatLxBI9gBaebbnrfifHhDYfgasaacH8akY=wiFfYdH8Gipec8Eeeu0xXdbba9frFj0=OqFfea0dXdd9vqai=hGuQ8kuc9pgc9s8qqaq=dirpe0xb9q8qiLsFr0=vr0=vr0dc8meaabaqaciaacaGaaeqabaqabeGadaaakeaacuWGtbWugaqcaaaa@2DEB@ for the data at hand (not shown, but see e.g. Figure 1 in [5]), which is essentially linear for genes with t-statistic |*t*| > 1, suggesting that the same general smoothing procedure is applicable.

### Performance on simulated data sets

We perform simulations with 10,000 genes per array, a proportion of truly nonDE genes *π*_0 _= 0.8, and two independent groups with *n *= 7 arrays per group. We combine three different levels of variance heterogeneity between genes with two different settings for the balance between up- and down-regulation, for a total of six different simulation scenarios:

1. Variances can be 'similar' (effectively the same) across genes, 'balanced', which allows for moderate differences in variance between genes, and 'variable', which allows large differences.

2. In the 'symmetric' case, roughly 50% of the DE genes are up- and down- regulated; in the 'asymmetric' case, only about 20% of all genes are down-regulated, the rest is up-regulated.

We have included the asymmetric scenario, because this is where ODP is expected to perform better than standard methods in a theoretical setting [5]. All expression values are assumed to follow a normal distribution; see Methods for further details of the simulation procedure.

For each scenario, we generate 100 data sets, for a total of 10^6 ^genes. For each procedure, the fdr values are computed by keeping track of the DE status of each gene, grouping the genes in intervals (1d) or grid cells (2d) based on their test statistic, and computing the percentage of false positives in each interval or cell.

**Figure 2:**
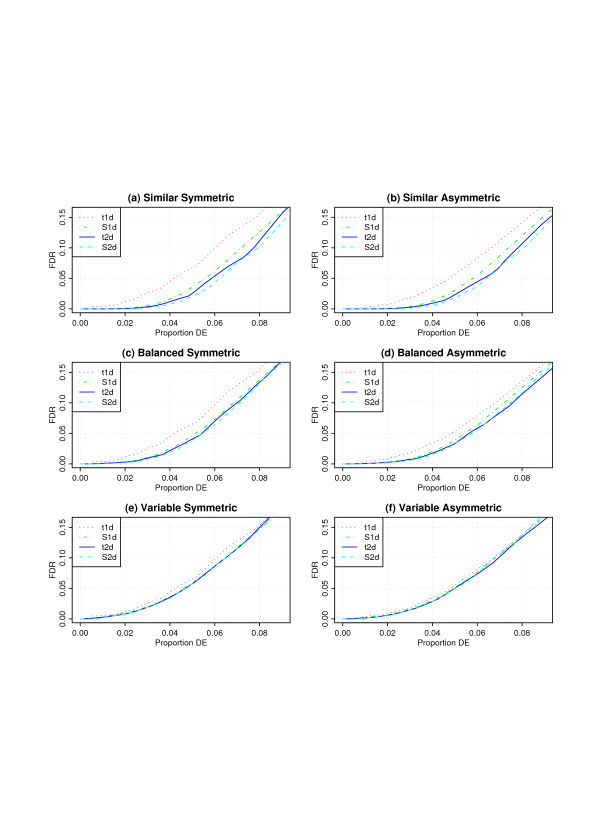
Operating characteristics of the four procedures for six simulated data sets. Each curve shows the true global FDR among the top-ranked genes for a procedure on the vertical axis as a function of the percentage of genes declared DE by this procedure on the horizontal axis. See text for description of the simulation scenarios.

In order to compare different fdr procedures, we summarize their results via operating characteristics (OC) curves: for each procedure, we sort the groups of genes as described above by their local fdr, and compute the corresponding global FDR as cumulative mean of the local fdrs from the smallest to the largest. This global FDR is then plotted against the cumulative percentage of genes in these intervals or grid cells. The resulting curve shows the true global FDR for a set of top-ranked genes as a function of the size of that set (as a percentage of the number of genes under study). The results for the different simulation scenarios and all four procedures are shown in Figure 2.

There is little or no difference in relative performance between the procedures under the symmetric and asymmetric scenarios in Figure 2. It is also clear that the differences in performance are most pronounced when the variances are similar, less so when the variances are balanced, and minor when the variances are highly variable. The ranking of the different procedures is consistent through all six scenarios: as expected, t1d has the worst performance; equally as expected, S1d does clearly better than t1d. Novel findings of this paper are that a) t2d does still better than S1d, and b) S2d improves over t2d, although only marginally.

### Performance on real datasets

We evaluate the performance of the different procedures on two real data sets:

• The BRCA data [13] contains 3,170 genes and was collected from 15 patients with hereditary breast cancer, who had mutations either of the BRCA1(*n *= 7) or the BRCA2 gene (*n *= 8).

• The Lymphoma data [14] contains 7,399 genes and was collected from 240 patients with diffuse large B-cell lymphoma, comprising *n*_1 _= 102 survivors and *n*_2 _= 138 non-survivors.

Here, the local fdr estimates are computed based on the mixture model (2). The estimate of *f *is computed by smoothing the histograms of the observed statistics, and similarly *f*_0 _from permuted test statistics. The permuted statistics are obtained from permutations of the group labels to generate the null distribution. Technically, we also need an estimate π^0
 MathType@MTEF@5@5@+=feaafiart1ev1aaatCvAUfKttLearuWrP9MDH5MBPbIqV92AaeXatLxBI9gBaebbnrfifHhDYfgasaacH8akY=wiFfYdH8Gipec8Eeeu0xXdbba9frFj0=OqFfea0dXdd9vqai=hGuQ8kuc9pgc9s8qqaq=dirpe0xb9q8qiLsFr0=vr0=vr0dc8meaabaqaciaacaGaaeqabaqabeGadaaakeaaiiGacuWFapaCgaqcamaaBaaaleaacqaIWaamaeqaaaaa@2F9A@ of the proportion of nonDE genes, although for the purpose of comparing the different procedures, it does not matter which estimate, as long as we use the same value for all procedures, see Methods. In fact, in comparing different FDR procedures, it is important that this parameter is set to the same value.

For each procedure, we rank the genes by their estimated fdr, and compute their estimated global FDR among the top-ranked genes as the cumulative mean of their local fdrs. The global FDR is then plotted as a function of the percentage of genes declared DE. For comparison purposes, we also include the FDR as computed by the EDGE software.

**Figure 3:**
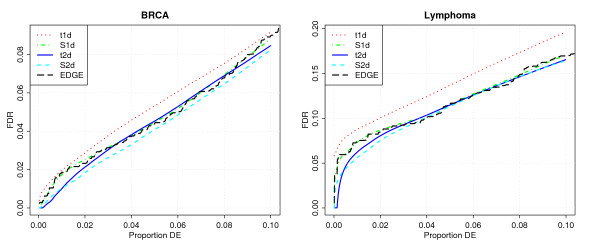
Operating characteristics of the four procedures and EDGE for the BRCA and Lymphoma data. Each curve shows the estimated global FDR among the top-ranked genes for a procedure on the vertical axis as a function of the percentage of genes declared DE by this procedure on the horizontal axis.

The resulting OC curves are shown in Figure 3. We get the same ranking as for the simulated data: t1d performs worst and is easily bettered by S1d; t2d performs better than S1d for the 2% most highly regulated genes, and is equivalent otherwise; S2d has a slight advantage over t2d on the BRCA data. Additionally, as a check that our implementation of ODP is correct, we are happy to see that EDGE and S1d yield virtually identical FDR curves.

**Figure 4:**
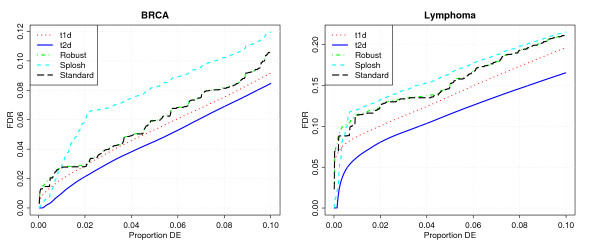
Operating characteristics of different procedures for the BRCA and Lymphoma data: t1d and t2d combine standard t-statistics with one- and two-dimensional local fdr as shown in Figure 3; 'Splosh' and 'robust' are the FDR procedures described in Pounds and Cheng (2004) and Pounds and Cheng (2006). The 'standard' method is described in Storey and Tibshirani (2003).

We [7] have previously compared t2d with other procedures such as SAM [11], Efron's modified t [10], and an empirical Bayes modification of the t-statistic [12]. To add more comparisons, we have run two procedures by Pounds and Cheng (Splosh [15] and robust FDR [16]) for the two real data sets. We use their own software, with a little modification so that we can specify the π^0
 MathType@MTEF@5@5@+=feaafiart1ev1aaatCvAUfKttLearuWrP9MDH5MBPbIqV92AaeXatLxBI9gBaebbnrfifHhDYfgasaacH8akY=wiFfYdH8Gipec8Eeeu0xXdbba9frFj0=OqFfea0dXdd9vqai=hGuQ8kuc9pgc9s8qqaq=dirpe0xb9q8qiLsFr0=vr0=vr0dc8meaabaqaciaacaGaaeqabaqabeGadaaakeaaiiGacuWFapaCgaqcamaaBaaaleaacqaIWaamaeqaaaaa@2F9A@ parameter to be the same as in the other procedures. The results in Figure 4 show both Splosh and robust FDR to perform worse than the other procedures. For these datasets, the robust FDR estimate coincides with the standard FDR estimate.

## Discussion

The main motivation for using the FDR has been that it offers a way of dealing with multiplicity that is less restrictive and more powerful than traditional p-value adjustments. The challenge is how to explicitly exploit the multiplicity by pooling information across genes in order to make the FDR even more powerful.

In the case of t1d, the test statistic is computed gene-by-gene and does not use information shared with other genes. Moderated t-statistics [10-12], which borrow strength across genes for estimating standard errors, are more powerful than simple t-statistics. The ODP appears to be the ultimate in combining information, where to some extent all genes contribute to the statistic for each other gene. The fdr2d approach on the other hand augments the grouping of genes based on individual test statistics by sub-grouping them based on their variability. In all cases we find that when there are few instances of genes with similar variability, the performance of the different methods tends to converge towards the simple t1d (Figures 2(e) and 2(f)).

From a practical point of view, it seems that the smoothing procedure underlying our implementation of fdr2d seems to work as well for the statistic logS^
 MathType@MTEF@5@5@+=feaafiart1ev1aaatCvAUfKttLearuWrP9MDH5MBPbIqV92AaeXatLxBI9gBaebbnrfifHhDYfgasaacH8akY=wiFfYdH8Gipec8Eeeu0xXdbba9frFj0=OqFfea0dXdd9vqai=hGuQ8kuc9pgc9s8qqaq=dirpe0xb9q8qiLsFr0=vr0=vr0dc8meaabaqaciaacaGaaeqabaqabeGadaaakeaacuWGtbWugaqcaaaa@2DEB@ in S2d as for the t-statistics in t2d, and arguably even better: when comparing Figures 1(c) and 1(d) in this paper with Figures 4(a) and 4(b) in [7], we find in the former less of a tendency to underestimate the fdr for genes with small effect sizes, as discussed in the previous paper.

At first glance, the empirical ODP statistic seems to rely on the assumption that the expression values for all genes are normally distributed. From a practical point of view, however, the empirical ODP procedure works even if the normal assumption does not hold, because it relies on the permutation algorithm. In this sense, the normal densities in (1) only represent a scoring function that exponentially downweights contributions from genes with different mean structure and/or large variability. However, the performance of the empirical ODP will depend on how precisely the normal assumption holds for the data at hand. Some loss of the optimality property in the real data applications is probably due to non-normality. But even in the simulations, the empirical ODP is not better than t2d. This can only mean that the presence of large number of nuisance parameters degrades the performance of ODP.

## Conclusion

The estimation of the nuisance parameters required to apply the ODP in practice makes the procedure described in [6] no longer optimal. We have shown in this paper that the combination of a conventional t-statistic with the standard error of the mean as described in [7] can outperform the empirical ODP. Further improvements can be made by combining the ODP test statistic with standard error information, but the gains are comparatively small.

The ODP procedure exploits similarities in the distribution for a collection of genes, for example similarity in variance. When variances between genes are dissimilar, there is little gain by the ODP compared to the standard t-statistic. One advantage of the ODP over the modified t-statistics is that the adaption is done automatically, without calculating a model-based or heuristic fudge factor for the denominator.

The computational demand of calculating the ODP statistic is a serious practical disadvantage: each density term *f*(*x*) or *g*(*x*) requires computation across the whole dataset, so a single ODP statistic already involves substantial computations. Doing this for the whole collection of genes and for repeated permutations of the group labels is an order of magnitude more laborious than the computation required for the standard statistics.

## Methods

### Simulation scenarios

Our model for simulating microarray data is based on the model described in [12]. We assume that the expression values for all *m *genes are normally distributed (possibly after suitable transformation), and that their variances s˜i2=(n1−1)si12+(n2−1)si22n1+n2−2.
 MathType@MTEF@5@5@+=feaafiart1ev1aaatCvAUfKttLearuWrP9MDH5MBPbIqV92AaeXatLxBI9gBaebbnrfifHhDYfgasaacH8akY=wiFfYdH8Gipec8Eeeu0xXdbba9frFj0=OqFfea0dXdd9vqai=hGuQ8kuc9pgc9s8qqaq=dirpe0xb9q8qiLsFr0=vr0=vr0dc8meaabaqaciaacaGaaeqabaqabeGadaaakeaacuWGZbWCgaacamaaDaaaleaacqWGPbqAaeaacqaIYaGmaaGccqGH9aqpdaWcaaqaaiabcIcaOiabd6gaUnaaBaaaleaacqaIXaqmaeqaaOGaeyOeI0IaeGymaeJaeiykaKIaem4Cam3aa0baaSqaaiabdMgaPjabigdaXaqaaiabikdaYaaakiabgUcaRiabcIcaOiabd6gaUnaaBaaaleaacqaIYaGmaeqaaOGaeyOeI0IaeGymaeJaeiykaKIaem4Cam3aa0baaSqaaiabdMgaPjabikdaYaqaaiabikdaYaaaaOqaaiabd6gaUnaaBaaaleaacqaIXaqmaeqaaOGaey4kaSIaemOBa42aaSbaaSqaaiabikdaYaqabaGccqGHsislcqaIYaGmaaGaeiOla4caaa@5161@σi2
 MathType@MTEF@5@5@+=feaafiart1ev1aaatCvAUfKttLearuWrP9MDH5MBPbIqV92AaeXatLxBI9gBaebbnrfifHhDYfgasaacH8akY=wiFfYdH8Gipec8Eeeu0xXdbba9frFj0=OqFfea0dXdd9vqai=hGuQ8kuc9pgc9s8qqaq=dirpe0xb9q8qiLsFr0=vr0=vr0dc8meaabaqaciaacaGaaeqabaqabeGadaaakeaaiiGacqWFdpWCdaqhaaWcbaGaemyAaKgabaGaeGOmaidaaaaa@30F0@ vary randomly between genes, following the scaled inverse of a *χ*^2^-distribution. Values are simulated for two groups of *n*_1 _and *n*_2 _arrays. Each gene *i *= 1, ... *m *to is selected randomly with probability *π*_0 _to be DE. For genes that are picked as nonDE, the mean value in both groups is set to zero; for genes that are selected as DE, the mean in the first group is set to zero, and the mean in the second group is drawn randomly from a normal distribution whose variance is proportional to the gene-specific variance σi2
 MathType@MTEF@5@5@+=feaafiart1ev1aaatCvAUfKttLearuWrP9MDH5MBPbIqV92AaeXatLxBI9gBaebbnrfifHhDYfgasaacH8akY=wiFfYdH8Gipec8Eeeu0xXdbba9frFj0=OqFfea0dXdd9vqai=hGuQ8kuc9pgc9s8qqaq=dirpe0xb9q8qiLsFr0=vr0=vr0dc8meaabaqaciaacaGaaeqabaqabeGadaaakeaaiiGacqWFdpWCdaqhaaWcbaGaemyAaKgabaGaeGOmaidaaaaa@30F0@.

In detail we proceed as follows for our simulations:

1. Initialize the design with *m *= 10,000 genes, proportion of nonDE genes *π*_0 _= 0.8, and two groups with *n*_1 _= *n*_2 _= 7.

2. For each gene *i *= 1, ... *m*, draw a gene-specific variance from

σi2~d0s02χd02,
 MathType@MTEF@5@5@+=feaafiart1ev1aaatCvAUfKttLearuWrP9MDH5MBPbIqV92AaeXatLxBI9gBaebbnrfifHhDYfgasaacH8akY=wiFfYdH8Gipec8Eeeu0xXdbba9frFj0=OqFfea0dXdd9vqai=hGuQ8kuc9pgc9s8qqaq=dirpe0xb9q8qiLsFr0=vr0=vr0dc8meaabaqaciaacaGaaeqabaqabeGadaaakeaaiiGacqWFdpWCdaqhaaWcbaGaemyAaKgabaGaeGOmaidaaOGaeiOFa43aaSaaaeaacqWGKbazdaWgaaWcbaGaeGimaadabeaakiabdohaZnaaDaaaleaacqaIWaamaeaacqaIYaGmaaaakeaacqWFhpWydaqhaaWcbaGaemizaqMaeGimaadabaGaeGOmaidaaaaakiabcYcaSaaa@3E83@

where χd02
 MathType@MTEF@5@5@+=feaafiart1ev1aaatCvAUfKttLearuWrP9MDH5MBPbIqV92AaeXatLxBI9gBaebbnrfifHhDYfgasaacH8akY=wiFfYdH8Gipec8Eeeu0xXdbba9frFj0=OqFfea0dXdd9vqai=hGuQ8kuc9pgc9s8qqaq=dirpe0xb9q8qiLsFr0=vr0=vr0dc8meaabaqaciaacaGaaeqabaqabeGadaaakeaaiiGacqWFhpWydaqhaaWcbaGaemizaqMaeGimaadabaGaeGOmaidaaaaa@31C8@ is a *χ*^2^-distribution with *d*_0 _degrees of freedom, and *d*_0 _and *s*_0 _are tuning parameters as described below.

3. For each gene *i *= 1,... *m*, determine randomly with probability *π*_0 _whether it is to be DE or not.

(a) In case of nonDE, set *μ*_1 _= *μ*_2 _= 0.

(b) In case of DE, set *μ*_1 _= 0 and draw *μ*_2 _randomly from

*D*_*i *_~ *N*(0, *v*_0_σi2
 MathType@MTEF@5@5@+=feaafiart1ev1aaatCvAUfKttLearuWrP9MDH5MBPbIqV92AaeXatLxBI9gBaebbnrfifHhDYfgasaacH8akY=wiFfYdH8Gipec8Eeeu0xXdbba9frFj0=OqFfea0dXdd9vqai=hGuQ8kuc9pgc9s8qqaq=dirpe0xb9q8qiLsFr0=vr0=vr0dc8meaabaqaciaacaGaaeqabaqabeGadaaakeaaiiGacqWFdpWCdaqhaaWcbaGaemyAaKgabaGaeGOmaidaaaaa@30F0@),

where is *v*_0 _is another tuning parameter.

i. In case of an asymmetric scenario, set the sign of *μ*_2 _to positive with probability 0.8, and to negative otherwise.

4. Simulate *n*_1 _and *n*_2 _values in the first and second group, respectively, following normal distributions

*X*_.*i*1 _~ *N*(*μ*_1_, σi2
 MathType@MTEF@5@5@+=feaafiart1ev1aaatCvAUfKttLearuWrP9MDH5MBPbIqV92AaeXatLxBI9gBaebbnrfifHhDYfgasaacH8akY=wiFfYdH8Gipec8Eeeu0xXdbba9frFj0=OqFfea0dXdd9vqai=hGuQ8kuc9pgc9s8qqaq=dirpe0xb9q8qiLsFr0=vr0=vr0dc8meaabaqaciaacaGaaeqabaqabeGadaaakeaaiiGacqWFdpWCdaqhaaWcbaGaemyAaKgabaGaeGOmaidaaaaa@30F0@),

*X*_.*i*2 _~ *N*(*μ*_2_, σi2
 MathType@MTEF@5@5@+=feaafiart1ev1aaatCvAUfKttLearuWrP9MDH5MBPbIqV92AaeXatLxBI9gBaebbnrfifHhDYfgasaacH8akY=wiFfYdH8Gipec8Eeeu0xXdbba9frFj0=OqFfea0dXdd9vqai=hGuQ8kuc9pgc9s8qqaq=dirpe0xb9q8qiLsFr0=vr0=vr0dc8meaabaqaciaacaGaaeqabaqabeGadaaakeaaiiGacqWFdpWCdaqhaaWcbaGaemyAaKgabaGaeGOmaidaaaaa@30F0@).

Following [12], we set the constants to s02
 MathType@MTEF@5@5@+=feaafiart1ev1aaatCvAUfKttLearuWrP9MDH5MBPbIqV92AaeXatLxBI9gBaebbnrfifHhDYfgasaacH8akY=wiFfYdH8Gipec8Eeeu0xXdbba9frFj0=OqFfea0dXdd9vqai=hGuQ8kuc9pgc9s8qqaq=dirpe0xb9q8qiLsFr0=vr0=vr0dc8meaabaqaciaacaGaaeqabaqabeGadaaakeaacqWGZbWCdaqhaaWcbaGaeGimaadabaGaeGOmaidaaaaa@3028@ = 4 and *v*_0 _= 2 in our simulations. The amount of variability of the gene-wise variances is controlled via the parameter *d*_0_: the three scenarios described in the Results section correspond to *d*_0 _= 1000 (similar variances across genes), *d*_0 _= 12 (balanced, with moderate differences between genes), and *d*_0 _= 2 (variable, with large variability in variances).

For each scenario, we then generate 100 data sets, for a total of 10^6 ^genes. For each procedure, the true local fdr of the genes is estimated from the known DE status of each simulated gene, simply as the proportion of false positives in each histogram interval or grid cell. This means specifically that no permutation, smoothing, or estimation of *π*_0 _is required.

### Real data sets

The permutation and smoothing approach used for estimating the fdr values for real data has been described in detail in [9] and [7]. The estimates π^0
 MathType@MTEF@5@5@+=feaafiart1ev1aaatCvAUfKttLearuWrP9MDH5MBPbIqV92AaeXatLxBI9gBaebbnrfifHhDYfgasaacH8akY=wiFfYdH8Gipec8Eeeu0xXdbba9frFj0=OqFfea0dXdd9vqai=hGuQ8kuc9pgc9s8qqaq=dirpe0xb9q8qiLsFr0=vr0=vr0dc8meaabaqaciaacaGaaeqabaqabeGadaaakeaaiiGacuWFapaCgaqcamaaBaaaleaacqaIWaamaeqaaaaa@2F9A@ for the proportion of nonDE genes are based on a mixture model for the observed distribution of t-statistics, consisting of one central and several non-central t-distributions; we have shown previously that the weight of the central t-distribution can be a less biased estimate of *π*_0 _in the presence of genes with small effects than the usual estimate based on the distribution of p-values ([17]). The same estimates have been used previously in [7].

The BRCA data set [13] was collected from patients with hereditary breast cancer who had mutations either of the BRCA1(*n *= 7) or the BRCA2 gene (*n *= 8). Expression was originally reported for 3,226 genes, but following [8], we removed 56 extremely variable genes and analysed only the remaining 3,170 genes. For all four procedures, we used π^0
 MathType@MTEF@5@5@+=feaafiart1ev1aaatCvAUfKttLearuWrP9MDH5MBPbIqV92AaeXatLxBI9gBaebbnrfifHhDYfgasaacH8akY=wiFfYdH8Gipec8Eeeu0xXdbba9frFj0=OqFfea0dXdd9vqai=hGuQ8kuc9pgc9s8qqaq=dirpe0xb9q8qiLsFr0=vr0=vr0dc8meaabaqaciaacaGaaeqabaqabeGadaaakeaaiiGacuWFapaCgaqcamaaBaaaleaacqaIWaamaeqaaaaa@2F9A@ = 0.61, and we evaluated 500 permutations of the group labels.

The Lymphoma data set [14] was collected from 240 patients with diffuse large B-cell lymphoma, *n*_1 _= 102 of whom survived the study period, and *n*_2 _= 138 of whom did not. We used all 7,399 genes reported in the original article. For all four procedures, we used π^0
 MathType@MTEF@5@5@+=feaafiart1ev1aaatCvAUfKttLearuWrP9MDH5MBPbIqV92AaeXatLxBI9gBaebbnrfifHhDYfgasaacH8akY=wiFfYdH8Gipec8Eeeu0xXdbba9frFj0=OqFfea0dXdd9vqai=hGuQ8kuc9pgc9s8qqaq=dirpe0xb9q8qiLsFr0=vr0=vr0dc8meaabaqaciaacaGaaeqabaqabeGadaaakeaaiiGacuWFapaCgaqcamaaBaaaleaacqaIWaamaeqaaaaa@2F9A@ = 0.59, and we evaluated 500 permutations of the group labels.

All expression values were logged prior to analysis.

### Software

Methods t1d and t2d are implemented in the R package OCplus, which is freely available at the Bioconductor website [18]. R code implementing S1d and S2d is available from the authors on request. EDGE, the official implementation of EODP described in [19], is available at [20].

## Competing interests

The author(s) declare that they have no competing interests.

## Authors' contributions

EP wrote computer programs, ran simulations and drafted the manuscript. AP wrote computer programs, ran data analysis and co-wrote the manuscript. SC co-wrote the manuscript. YP conceived the study and drafted the manuscript. All authors read and approved the final manuscript.

## References

[B1] Allison DB, Cui X, Page GP, Sabripour M (2006). Microarray data analysis: from disarray to consolidation and consensus. Nat Rev Genet.

[B2] Datta S, Datta S (2005). Empirical Bayes screening of many p-values with applications to microarray studies. Bioinformatics.

[B3] Benjamini Y, Hochberg Y (1995). Controlling the false discovery rate – A practical and powerful approach to multiple testing. J Roy Stat Soc B.

[B4] Choe S, Boutros M, Michelson A, Church G, Halfon M (2005). Preferred analysis methods for Affymetrix GeneChips revealed by a wholly defined control dataset. Genome Biology.

[B5] Storey JD (2005). The Optimal Discovery Procedure: A New Approach to Simultaneous Significance Testing. UW Biostatistics Working Paper Series Working Paper 259.

[B6] Storey JD, Dai JY, Leek JT (2005). The Optimal Discovery Procedure for Large-Scale Significance Testing, with Applications to Comparative Microarray Experiments. UW Biostatistics Working Paper Series Working Paper 260.

[B7] Ploner A, Calza S, Gusnanto A, Pawitan Y (2006). Multidimensional local false discovery rate for microarray studies. Bioinformatics.

[B8] Storey JD, Tibshirani R (2003). Statistical significance for genomewide studies. Proc Natl Acad Sci USA.

[B9] Efron B, Tibshirani R, Storey J, Tusher V (2001). Empirical Bayes Analysis of a Microarray Experiment. J Am Stat Soc.

[B10] Efron B, Tibshirani R, Chu GossGV (2000). Microarrays and their use in a comparative experiment. Technical report.

[B11] Tusher V, Tibshirani R, Chu G (2001). Significance analysis of microarrays applied to the ionizing radiation response. PNAS.

[B12] Smyth G (2004). Linear Models and Empirical Bayes Methods for Assessing Differential Expression in Microarray Experiments. Statistical Applications in Genetics and Molecular Biology.

[B13] Hedenfalk I, Duggan D, Chen Y, Radmacher M, Bittner M, Simon R, Meltzer P, Gusterson B, Esteller M, Kallioniemi OP, Wilfond B, Borg A, Trent J (2001). Gene-expression profiles in hereditary breast cancer. N Engl J Med.

[B14] Rosenwald A, Wright G, Chan WC, Connors JM, Campo E, Fisher RI, Gascoyne RD, Muller-Hermelink HK, Smeland EB, Giltnane JM, Hurt EM, Zhao H, Averett L, Yang L, Wilson WH, Jaffe ES, Simon R, Klausner RD, Powell J, Duffey PL, Longo DL, Greiner TC, Weisenburger DD, Sanger WG, Dave BJ, Lynch JC, Vose J, Armitage JO, Montserrat E, LApez-Guillermo A, Grogan TM, Miller TP, LeBlanc M, Ott G, Kvaloy S, Delabie J, Holte H, Krajci P, Stokke T, Staudt LM, Project LMP (2002). The use of molecular profiling to predict survival after chemotherapy for diffuse large-B-cell lymphoma. N Engl J Med.

[B15] Pounds S, Cheng C (2004). Improving false discovery rate estimation. Bioinformatics.

[B16] Pounds S, Cheng C (2006). Robust estimation of the false discovery rate. Bioinformatics.

[B17] Pawitan Y, Murthy KRK, Michiels S, Ploner A (2005). Bias in the estimation of false discovery rate in microarray studies. Bioinformatics.

[B18] Bioconductor. http://www.bioconductor.org.

[B19] Leek JT, Monsen E, Dabney AR, Storey JD (2006). EDGE: extraction and analysis of differential gene expression. Bioinformatics.

[B20] EDGE. http://www.biostat.washington.edu/software/jstorey/edge.

